# The Relationship of Sensory Profiles and Peripheral Biomarkers with Obesity and Eating Styles in Adolescence

**DOI:** 10.3390/nu17243923

**Published:** 2025-12-15

**Authors:** Nagihan Erdog Sahin, Nihal Hatipoglu, Didem Barlak Keti, Esra Demirci, Meda Kondolot

**Affiliations:** 1 Social Pediatrics Unit, Department of Pediatrics, Kayseri City Hospital, 38080 Kayseri, Turkey; 2 Department of Pediatric Endocrinology, Faculty of Medicine, Erciyes University, 38039 Kayseri, Turkey; nihalhatipoglu@yahoo.com; 3 Department of Biochemistry, Faculty of Medicine, Erciyes University, 38039 Kayseri, Turkey; dbarlakketi@erciyes.edu.tr; 4 Department of Child and Adolescent Psychiatry, Faculty of Medicine, Erciyes University, 38039 Kayseri, Turkey; esra_z_d_r@hotmail.com; 5 Social Pediatrics Unit, Department of Pediatrics, Ankara Etlik City Hospital, 06170 Ankara, Turkey; medakondolot@gmail.com

**Keywords:** obesity, adolescents, eating behaviors, sensory profiles, leptin, ghrelin, cortisol

## Abstract

Background/Objectives: The increasing prevalence of obesity among children and adolescents is alarming, and the obesogenic environment is considered a major contributing factor to this public health concern. Nevertheless, individuals exposed to the same obesogenic environment exhibit considerable variability in their body weight and eating behaviors. Therefore, this study investigated the relationships between eating behaviors, sensory processing profiles, and peripheral biomarkers in the context of adolescent obesity. Methods: A total of 99 adolescents aged 11–18 years (51 in the obese group and 48 in the control group) were enrolled in the study. Blood and saliva samples were obtained from participants. All participants also completed a Sociodemographic Information Form, the Adolescent/Adult Sensory Profile (A/ASP), and the Dutch Eating Behavior Questionnaire (DEBQ). Results: No statistically significant differences were found between the groups in any of the sensory processing quadrants (*p* > 0.05). A moderate positive correlation was observed between Sensory Sensitivity (A/ASP) and Emotional Eating (DEBQ) (r = 0.442, *p* < 0.001), whereas no other associations between A/ASP quadrants and DEBQ subscales reached statistical significance after adjustment for multiple comparisons. After controlling for the effects of sex, BMI and physical activity, plasma leptin, ghrelin, and salivary cortisol levels were not significantly associated with restrained, emotional, or external eating behaviors (*p* > 0.05). Conclusions: Obese and control group adolescents exhibited similar sensory processing profiles, yet higher sensory sensitivity scores were associated with greater emotional eating. These findings suggest that sensory sensitivity may be relevant for understanding emotional eating in adolescence; however, longitudinal research is required to determine whether this association is causal and to clarify the processes underlying it.

## 1. Introduction

Obesity, recognized as a chronic, complex, and multifactorial disease, has been steadily increasing over the past five decades, reaching pandemic proportions worldwide [1]. The increasing prevalence of obesity among children and adolescents is of particular concern. According to estimates by the World Obesity Federation (2019), approximately 254 million individuals aged 5–19 are expected to be living with obesity by 2030 [2]. Secular trends have drawn significant attention to the etiological factors implicated in this epidemiological rise in obesity.

Obesity is primarily attributed to an imbalance in energy homeostasis, with the obesogenic environment—characterized by excessive energy intake and reduced physical activity—being considered a major contributing factor. Nevertheless, individuals exposed to the same obesogenic environment display considerable variability in body weight and in their eating behavior responses [3,4]. Eating behaviors (i.e., eating styles) have emerged as important determinants of body weight from childhood onward [3,5], and emotional, external, and restrained eating have each been associated with obesity [4,6,7]. Building on this perspective, the present study focuses on two domains—sensory processing profiles and peripheral biomarkers—whose role in adolescent obesity and eating styles has not been extensively investigated.

Sensory processing refers to the way the central nervous system perceives, discriminates, and regulates input from eight fundamental sensory systems—olfactory, gustatory, tactile, visual, auditory, vestibular, proprioceptive, and interoceptive—and provides the basis for motor, emotional, behavioral, and adaptive responses [8]. Certain regions of the orbitofrontal cortex have been implicated in subjective evaluations of the taste, smell, and appearance of food, thereby linking sensory processing with the regulation of food intake. Eating is inherently a sensory activity, and dietary intake and eating behaviors can therefore be influenced by sensory processing mechanisms [9]. Importantly, adolescence is a neurodevelopmental period during which sensory integration, reward processing, and executive functions undergo distinct maturation, and each of these changes can shape adolescents’ eating behaviors. Although several studies have examined the relationship between sensory processing and BMI [10,11], the association between sensory processing and eating styles—emotional, external, and restrained—has not yet been comprehensively investigated in adolescents.

Eating behaviors are closely linked to appetite, which is regulated through the interaction of homeostatic and hedonic mechanisms. Leptin (anorexigenic) and ghrelin (orexigenic) are key neuropeptide hormones that regulate energy balance through homeostatic pathways and also influence reward-related aspects of eating [12,13,14,15]. Cortisol, the end-product of hypothalamic–pituitary–adrenal (HPA) axis activation, plays an important role in stress responses and has been implicated in stress-related obesity and appetite regulation [16]. Morning salivary cortisol may be used as a biomarker, as it may reflect long-term HPA axis dysregulation that could plausibly be associated with trait eating behaviors like emotional eating. Nevertheless, the contribution of these appetite- and obesity-related hormones to specific eating styles—such as emotional, external, and restrained eating—during adolescence remains unclear [17,18,19,20].

To address these gaps in the literature, this study examined the associations of sensory processing profiles and peripheral biomarkers (leptin, ghrelin, and cortisol) with emotional, external, and restrained eating styles in the context of adolescent obesity. The findings may provide preliminary insights into how sensory and hormonal factors relate to eating behaviors during adolescence, a critical period for the formation of long-term eating patterns, and may help guide future research in this area.

## 2. Materials and Methods

### 2.1. Study Design, Participants, and Procedure

This cross-sectional, comparative study was conducted between February 2022 and March 2023 at the Departments of Pediatric Endocrinology and Social Pediatrics, Children’s Hospital, Faculty of Medicine, Erciyes University (Kayseri, Türkiye). Using G*Power software (version 3.1), we conducted an a priori sample size calculation with an alpha level of 0.05, a target statistical power of 0.85, and an anticipated moderate effect size of 0.395 for group comparisons on behavioral measures. A total of 99 adolescents aged 11–18 years were ultimately enrolled, including 51 in the obese group (OB) and 48 in the control group (CG), which met the required sample size based on this a priori power analysis.

The obese group comprised adolescents with exogenous obesity who had not previously received treatment for obesity. Obesity was defined as a body mass index z-score (BMIz) ≥ +2 SD for age and sex, according to the World Health Organization (WHO) growth reference standards for individuals aged 5–19 years. Exclusion criteria included: (1) syndromic or endocrinological disorders causing obesity; (2) use of medications associated with weight gain (e.g., glucocorticoids, antidepressants, antipsychotics, antihistamines, valproic acid, or other anticonvulsants); (3) neurological or psychiatric disorders (e.g., neurodevelopmental disorders, eating disorders); and (4) any diagnosed chronic medical condition. The control group consisted of adolescents with normal weight, defined as having a BMIz between −2 SD and +1 SD (−2 SD ≤ BMIz < +1 SD). Exclusion criteria included any psychiatric disorder, chronic medical disease, or regular medication use.

Adolescents in the control group were evaluated by a pediatrician, and those in the obese group by a pediatric endocrinologist. All participants were additionally evaluated by an experienced child and adolescent psychiatrist using the Turkish version of the Schedule for Affective Disorders and Schizophrenia for School-Age Children—Present and Lifetime Version (K-SADS-PL-T) [21]. Adolescents who met criteria for any psychiatric disorder on this structured interview were excluded from the study. Anthropometric measurements were obtained, after which saliva samples were collected for cortisol assays and venous blood samples were drawn for leptin and ghrelin analyses. Participants also completed a Sociodemographic Information Form, the Adolescent/Adult Sensory Profile, and the Dutch Eating Behavior Questionnaire.

This study was approved by the Ethics Committee for Clinical Research of Erciyes University (approval no: 2022/87, 9 February 2022) and conducted in accordance with the principles of the Declaration of Helsinki. Written informed consent was obtained from all adolescents and their parents/guardians prior to enrollment. The study was supported by the Scientific Research Projects Unit of Erciyes University (project no: TDK-2022-12042).

### 2.2. Measures

#### 2.2.1. Sociodemographic Characteristics

A sociodemographic information form, designed to collect data on adolescents and their parents’ sociodemographic characteristics and lifestyle factors, was completed jointly by the participants and their parents. For adolescents, the form included age (day/month/year), sex (female/male), body weight, height, sleep duration (≤8 h, >8 h), presence of late bedtime and/or irregular sleep–wake patterns (yes/no), screen time (≤3 h, >3 h), and physical activity level (inactive/less active, active/very active). Late bedtimes (after midnight) and/or irregular sleep–wake schedules were classified as late or irregular sleep. Participants who self-identified as “inactive” or “less active” were categorized as physically inactive, whereas those who reported being “active” or “very active” were categorized as physically active. Family-related variables included household income level (expenses > income, income = expenses, income > expenses) and parental education level (primary or less, secondary, high school or above).

#### 2.2.2. Anthropometric Measurements and Body Mass Index

Height and weight measurements were obtained in the morning after an overnight fast, with participants wearing light clothing and no shoes, under standardized conditions. A digital stadiometer and scale (Densi Industrial Weighing Systems) were used, with a precision of 0.1 kg for weight and 0.5 cm for height. Body mass index (BMI) and BMI z-scores (BMIz) were calculated for each participant using the World Health Organization (WHO) standardized reference values for age and sex in individuals aged 5–19 years.

#### 2.2.3. Adolescent/Adult Sensory Profile (A/ASP)

The sensory profiles of the participants were assessed using the Adolescent/Adult Sensory Profile (A/ASP), a 60-item self-report questionnaire for individuals aged 11–65+ that measures the frequency of responses to everyday sensory experiences [22]. Items were rated on a 5-point Likert scale, where 1 = ‘almost never’, 2 = ‘seldom’, 3 = ‘occasionally’, 4 = ‘frequently’, and 5 = ‘almost always’. The instrument evaluates sensory processing within four quadrants based on Dunn’s model: Low Registration (high thresholds, reduced or delayed responses), Sensation Seeking (actively engaging in sensory input), Sensory Sensitivity (low thresholds, heightened responsiveness), and Sensation Avoiding (purposefully limiting sensory input). Items cover everyday domains such as taste/smell, movement, vision, touch, hearing, and activity level. Scores for each quadrant were summed, and participants were classified using the normative data provided in the manual for their age group (11–17 years in this study) as ‘much less than most people,’ ‘less than most people,’ ‘similar to most people,’ ‘more than most people,’ or ‘much more than most people’ [22,23].

The Turkish adaptation of the A/ASP was validated by Üçgül et al. [24], demonstrating acceptable reliability and validity. Reported Cronbach’s alpha values were 0.82 for Low Registration, 0.79 for Sensation Seeking, 0.81 for Sensory Sensitivity, and 0.66 for Sensation Avoiding, with test–retest reliability ranging between r = 0.67 and r = 0.82. In the present sample, internal consistency coefficients for the A/ASP subscales were acceptable, with Cronbach’s alpha values of 0.776 for Low Registration, 0.704 for Sensation Seeking, 0.778 for Sensory Sensitivity, and 0.750 for Sensation Avoiding. The total scale also showed good overall reliability (α = 0.895).

#### 2.2.4. Dutch Eating Behavior Questionnaire (DEBQ)

The Dutch Eating Behavior Questionnaire (DEBQ) is a 33-item self-report instrument developed by van Strien et al. [7] to assess eating behaviors. The questionnaire includes three subscales: Emotional Eating, External Eating, and Restrained Eating. Items are rated on a 5-point Likert scale ranging from 1 (never) to 5 (very often), with higher scores on each subscale indicating a greater tendency toward that eating behavior. The Turkish adaptation of the DEBQ was validated by Bozan et al. [25], demonstrating excellent reliability, with Cronbach’s alpha values of 0.92 for Emotional Eating, 0.96 for Restrained Eating, and 0.90 for External Eating. In the present sample, internal consistency was also high, with Cronbach’s alpha values of 0.947 for Emotional Eating, 0.918 for Restrained Eating, 0.821 for External Eating, and 0.890 for the total DEBQ score.

#### 2.2.5. Biochemical Analyses

Blood and saliva samples were collected between 8:30 and 9:30 a.m. after an overnight fast. Saliva samples were obtained before venipuncture. Salivary cortisol was assessed because saliva contains biologically active cortisol [26] and can be collected noninvasively, thus minimizing the possibility that the sampling procedure would elicit an acute stress response. Parents and adolescents received written and verbal instructions on the procedure, including refraining from food, beverages, toothbrushing, or exercise for at least 30 min prior to sampling. Salivary cortisol was collected using Salivette^®^ devices (Sarstedt, Germany). Adolescents placed the cotton roll in their mouths for approximately 2 min until saturated, then returned it directly to the tube without hand contact. Tubes were centrifuged at 2000× *g* for 10 min at 4 °C, and supernatant saliva was stored at −80 °C until assay. Cortisol concentrations were measured using electrochemiluminescence immunoassay (ECLIA) on a Roche Cobas e801 analyzer.

Venous blood samples (5–6 mL) were drawn from the median antecubital vein for leptin and ghrelin measurement. After centrifugation at 2000× *g* for 10 min at 4 °C, serum aliquots were stored at −80 °C. Serum leptin and ghrelin levels were quantified using ELISA kits (Bostonchem, Cambridge, MA, USA; leptin, catalog no. BLS-1160Hu; ghrelin, catalog no. BLS-1943Hu) according to the manufacturer’s instructions. Absorbance was read at 450 nm using a microplate ELISA reader (BioTek Instruments, Winooski, VT, USA; ELx800). Because of sample integrity and feasibility constraints, serum leptin and ghrelin analyses were performed in 44 participants per group.

### 2.3. Statistical Analysis

The normality of data distribution was assessed using the Shapiro–Wilk test, histograms, and Q–Q plots. Homogeneity of variance was evaluated with Levene’s test. Quantitative variables were compared between groups using independent-samples *t*-tests or Mann–Whitney U tests, and categorical variables were analyzed using Pearson’s χ^2^ test. Correlations were examined with Pearson or Spearman analyses, depending on data distribution. Hierarchical multiple regression models were used to assess the effects of leptin, ghrelin, and cortisol on eating behaviors, controlling for sex and BMI. Regression assumptions (normality, homoscedasticity, and multicollinearity) were checked and met. Internal consistency (Cronbach’s alpha) was calculated for the A/ASP and DEBQ subscales in the present sample to evaluate scale reliability. Analyses were conducted with SPSS version 27.0 (IBM Corp., Armonk, NY, USA), and statistical significance was set at *p* < 0.05.

## 3. Results

### 3.1. Sample Characteristics of the Groups

Comparisons of sociodemographic characteristics, screen time, physical activity level, sleep patterns, and parental education and income between the OB and CG are presented in Table 1. The OB and CG did not differ significantly with respect to age, sex, height, screen time, late and/or irregular sleep, sleep duration, parental education levels, or household income (*p* > 0.05; Table 1). However, a significantly greater proportion of adolescents in the OB were classified as physically inactive compared with those in the CG (*p* < 0.001).

### 3.2. Comparison of A/ASP and DEBQ Scores Between the Groups

Participants’ sensory processing was assessed using the Adolescent/Adult Sensory Profile (A/ASP). Mean raw scores for the Low Registration, Sensation Seeking, Sensory Sensitivity, and Sensation Avoiding quadrants are presented in Table 1. There were no statistically significant differences between the OB and CG in any of the sensory processing quadrants (*p* > 0.05; Table 1). Group distributions across the quadrants are shown in Table 2, indicating that in both groups, at least half of the adolescents were classified as ‘similar to most people’ in each domain.

Eating behaviors were assessed using the Dutch Eating Behavior Questionnaire (DEBQ). A significant group difference was found in the External Eating subscale (*p* = 0.024), with higher mean scores in the OB compared with the CG. There were no significant group differences for the Restrained or Emotional Eating subscales (*p* > 0.05; Table 1). When DEBQ scores were analyzed by sex, emotional eating scores were significantly higher in females than in males in the overall sample (*p* = 0.021; Supplementary Materials, Table S1). Moreover, within the OB, female adolescents had significantly higher emotional eating scores than male adolescents (*p* = 0.047; Supplementary Materials, Figure S1).

### 3.3. Correlations Between A/ASP and DEBQ Scores

In the combined analysis of participants from both the OB and CG, a moderate positive correlation was found between Sensory Sensitivity (A/ASP) and Emotional Eating (DEBQ) (*p* < 0.01, r = 0.442). A weak but statistically significant positive correlation was also observed between External Eating (DEBQ) and Sensory Sensitivity (A/ASP) (*p* = 0.016, r = 0.242). There were no additional significant correlations between A/ASP quadrants and DEBQ subscales (*p* > 0.05; Table 3). To account for multiple testing, the Benjamini–Hochberg false discovery rate (BH-FDR) procedure was applied to the correlation analyses. After FDR adjustment, the weak association previously observed between Sensory Sensitivity and External Eating (r = 0.242, *p* = 0.016) did not remain statistically significant, indicating that this finding may have reflected a chance association. In contrast, the moderate correlation between Sensory Sensitivity and Emotional Eating (r = 0.442, *p* < 0.01) remained significant after correction. No other correlations between A/ASP quadrants and DEBQ subscales were statistically significant following FDR adjustment.

### 3.4. Findings from Biochemical Analyses

Blood and saliva samples were obtained from all participants (51 OB, 48 CG). Plasma leptin and ghrelin levels were analyzed in 44 adolescents per group, while salivary cortisol levels were measured in all participants. Median plasma leptin concentrations were 245.5 pg/mL (177.0–387.8) in the CG and 275.0 pg/mL (176.8–495.5) in the OB, with no significant difference between groups (Mann–Whitney U test, *p* = 0.679). Median plasma ghrelin concentrations were 1927.0 pg/mL (679.8–3002.0) in the CG and 1177.0 pg/mL (483.3–2079.0) in the OB, with no significant difference between groups (Mann–Whitney U test, *p* = 0.108). Median salivary cortisol concentrations did not differ significantly between the CG and OB (CG: 0.2 µg/dL [0.2–0.3]; OB: 0.2 µg/dL [0.1–0.3]; *p* = 0.774) (See Supplementary Materials, Table S2).

### 3.5. Hierarchical Multiple Regressions

To examine the effects of leptin, ghrelin, and cortisol levels on emotional, external, and restrained eating behaviors, hierarchical multiple regression analyses were performed while controlling for sex, BMI, and physical activity (PA). Control variables (sex, BMI, PA) were entered in Block 1, followed by the biochemical variables (leptin, ghrelin, and cortisol) in Block 2.

### 3.6. Emotional Eating

In Block 1, sex, BMI, and PA explained 10% of the variance (F = 3.234, *p* = 0.026). In Block 2, after controlling for sex, BMI, and PA, the addition of leptin, ghrelin, and cortisol did not explain any additional significant variance in emotional eating, and none of the biomarkers were significantly associated with the outcome (*p* > 0.05; Table 4).

### 3.7. External Eating

In Block 1, BMI, sex, and PA explained 10% of the variance (F = 3.211, *p* = 0.027). In Block 2, after controlling for sex, BMI, and PA, the addition of leptin, ghrelin, and cortisol did not explain any additional significant variance in external eating, and none of the biomarkers were significantly associated with the outcome (*p* > 0.05; Table 5).

### 3.8. Restrained Eating

After controlling for sex, BMI, and PA, the addition of leptin, ghrelin, and cortisol levels was not significantly associated with restrained eating, and these biomarkers did not explain any additional significant variance in the outcome (*p* > 0.05; Table 6).

## 4. Discussion

This study initially investigated the relationship between sensory processing profiles, obesity, and eating styles in adolescents. The findings indicated that obese and normal-weight adolescents exhibited similar sensory processing profiles when assessed using the Adolescent/Adult Sensory Profile (A/ASP). In both groups, at least 50% of participants demonstrated sensory processing patterns comparable to those of the general population across all four quadrants: Low Registration, Sensation Seeking, Sensory Sensitivity, and Sensation Avoiding.

Additionally, initial correlation analyses revealed a moderate positive association between sensory sensitivity and emotional eating, as well as a weak but statistically significant association between sensory sensitivity and external eating. However, after adjusting for multiple comparisons, only the association with emotional eating remained statistically significant. This suggests that individuals with heightened sensory sensitivity may be more prone to emotional eating. By contrast, the weak association between sensory sensitivity and external eating did not survive correction for multiple comparisons, indicating that it may have reflected a chance finding rather than a robust effect. To our knowledge, this is the first study to examine associations between sensory processing profiles and eating behaviors in adolescents and to demonstrate a reliable relationship between sensory sensitivity and emotional eating in this age group.

These findings are partly consistent with previous research. A cross-sectional study of children aged 3 to 7 years assessing the relationship between BMI and sensory processing found, similar to the present study, no association between child overweight and obesity and the prevalence of atypical sensory outcomes [11]. However, a one-point increase in BMI was associated with a higher prevalence of atypical tactile sensitivity [11]. In a study conducted with adult women, participants were classified as having either high or low sensory sensitivity based on their sensory sensitivity quadrant scores on the Adolescent/Adult Sensory Profile (A/ASP), and their chocolate intake was measured during an experimental task [27]. Participants with high sensory sensitivity consumed significantly more chocolate, and a positive association was observed between sensory sensitivity scores and emotional eating scores [27]. Consistent with these findings, the present study found a positive association between sensory sensitivity and emotional eating, which is in line with previous research.

Most of the existing studies on sensory processing have focused on younger children and those with feeding difficulties, such as food refusal or food selectivity [10,28,29,30,31,32,33]. Another group in which the relationship between sensory processing and feeding has been frequently investigated is children with developmental conditions such as autism spectrum disorder (ASD) [34,35,36]. Feeding problems, particularly food selectivity, are highly prevalent among children with ASD, and one plausible explanation for this elevated prevalence is the heightened sensory sensitivity commonly observed in this population [34,37]. This association between sensory sensitivity and food selectivity has been reported not only in individuals with ASD but also at subclinical levels in the general population [37]. High sensory sensitivity has been associated with lower fruit and vegetable intake, and children with atypical tactile and taste/smell sensitivity have been reported to consume fewer Mediterranean foods such as vegetables, fruits, fish, legumes, grains, and olive oil [28,29]. Here, it has been proposed that sensory sensitivity may make some foods more aversive because of their sensory characteristics, while at the same time increasing the desirability of other foods [28,29]. In this context, the anticipated positive association between sensory sensitivity and external eating—eating elicited by external cues such as the sight, smell, or taste of food—was not detected in our sample. Given the limited sample size, this null finding should be viewed cautiously.

The positive association between sensory sensitivity and emotional eating identified in our study suggests that, as sensory sensitivity increases, individuals may be more likely to eat in response to negative emotional states, independent of internal physiological cues such as hunger and satiety. Individuals with elevated sensory sensitivity may experience negative emotions differently or may employ alternative coping strategies—such as emotional eating—to regulate these emotions. Indeed, sensory processing patterns have been shown to influence how individuals experience and cope with anxiety [38], which may explain why emotional eating serves as one of the ways in which adolescents with heightened sensory sensitivity respond to stressful or anxiety-provoking situations. Drawing definitive conclusions about how sensory sensitivity influences emotional eating would extend beyond the scope of the current findings; however, we believe that these data provide a solid foundation for further investigation into the role of sensory processing in emotional eating.

As a secondary focus, the present study explored the relationships between key appetite- and obesity-related biomarkers (serum leptin, serum ghrelin, and salivary cortisol) and eating behaviors, as well as their potential regulatory roles in shaping these behaviors. After controlling for the effects of sex, BMI, and physical activity; plasma leptin concentrations were not associated with restrained, emotional, or external eating behaviors. Previous research examining the role of leptin in eating behaviors has produced heterogeneous findings [39,40,41,42]. In a study of school-aged children (5–12 years), hyperleptinemia was reported to develop in girls in response to stress, and emotional eating increased when both cortisol and leptin levels were elevated [39]. In adults, higher leptin levels were found to be associated with uncontrolled eating, and this relationship was moderated by insulin sensitivity [40]. In the present study, while sex and BMI significantly predicted emotional eating, plasma leptin concentrations were not directly associated with this behavior.

In addition to leptin, ghrelin was examined in relation to eating behaviors. After controlling for the effects of sex, BMI, and physical activity; plasma ghrelin concentrations were not significantly associated with restrained, emotional, or external eating behaviors. In a study conducted in adolescents aged 8–16 years with obesity using a design similar to the present research, individuals with higher ghrelin concentrations exhibited lower scores for restrained eating—a finding that differs from our results [42]. In the same study, consistent with our findings, no associations were observed between leptin levels and eating behaviors [42]. Previous research investigating the relationship between ghrelin and restrained eating has yielded heterogeneous findings [43,44].

Similarly, salivary cortisol levels were not associated with emotional, external, or restrained eating behaviors after controlling for sex, BMI, and physical activity. This finding is consistent with the investigation that found no relationship between salivary cortisol levels and emotional eating in preschool children [45]. Salivary cortisol is widely used as a stress biomarker in eating behavior research because it reflects biologically active, free cortisol. However, the literature presents conflicting evidence: while some studies have reported positive associations between cortisol levels, stress, and emotional eating, others have not. In an experimental study [46] used salivary cortisol was used to assess acute stress responses and found that individuals with stronger stress reactions engaged in more unhealthy and emotional eating. These inconsistencies between studies are thought to be largely due to methodological differences. However, these null findings between hormones and eating behaviors should be interpreted cautiously. The study was not specifically powered to detect biomarker–behavior associations, and the use of single-time-point hormonal measurements, combined with the modest sample size and the inherent biological variability of leptin, ghrelin, and cortisol, may have limited our ability to detect subtle physiological relationships.

The present study has several methodological strengths compared with previous research. These include the use of a sample within a narrow age range, the inclusion of both adolescents with obesity and those with normal BMI, and statistical adjustments to control for potential confounding effects of sex and BMI. Together, these features provide a more rigorous assessment of the relationships between hormonal profiles and eating behaviors.

This study has several limitations. First, the sample size may be considered a limitation. Although the number of participants provided sufficient statistical power to detect differences between groups, it may still have limited the ability to identify smaller or more subtle effects. Furthermore, the study was not powered to detect associations between appetite-related biomarkers and behavioral outcomes. The sample size calculation was based on identifying group differences in behavioral measures, and the analyses involving leptin, ghrelin, and cortisol should therefore be considered exploratory. Given the modest sample size, the biological variability of hormonal measures, the reliance on single-time-point morning sampling, potential assay-related variability, and the possibility of residual confounding, the absence of statistically significant associations should be interpreted cautiously. Second, although all scales used in the study demonstrated validity and reliability, the use of self-report questionnaires introduces the possibility of recall bias and socially desirable responding. In addition to these limitations, sensory processing in this study was assessed using the Adolescent/Adult Sensory Profile, a global measure of sensory responsivity across daily life contexts. Although the A/ASP includes items related to taste and smell, it may not capture subtle chemosensory sensitivities or food-specific sensory responses. Therefore, our findings should be interpreted as reflecting general sensory processing patterns rather than narrowly defined taste or smell sensitivities, and direct comparisons with studies using food-specific sensory instruments should be made with caution. Additionally, the A/ASP is ideally completed within a clinical evaluation by an occupational therapist trained in sensory integration; in our study, it was used as a questionnaire-based measure only, which may have constrained the clinical interpretability of the sensory findings. Lastly, the cross-sectional design of the study does not allow for the establishment of causal relationships between eating behaviors and hormonal measures (leptin, ghrelin, and cortisol), underscoring the need for future longitudinal research to clarify these associations.

Despite these limitations, this study makes an important contribution to understanding the interplay between sensory characteristics, circulating hormones, obesity, and eating behaviors in adolescents. Future longitudinal or experimental studies with larger sample sizes and repeated hormonal measurements will be essential for further elucidating the complex nature of these relationships.

## 5. Conclusions

This study provides preliminary insights into the interplay between sensory processing, eating behaviors, and obesity in adolescence. Although obese and normal-weight adolescents showed largely similar sensory processing profiles, heightened sensory sensitivity was associated with greater emotional eating. The initially observed weak association with external eating did not remain significant after correction for multiple comparisons, suggesting that it may not reflect a robust effect. No significant associations were found between appetite-related biomarkers (leptin, ghrelin, and cortisol) and restrained, emotional, or external eating after adjustment for sex and BMI. These null findings should be interpreted with caution, as single-time-point biomarker assessments, the modest sample size, and the timing of sampling may have limited the ability to detect subtle physiological associations. Taken together, these findings suggest that sensory sensitivity may be a relevant factor in adolescent eating patterns, while the relative contributions of sensory and biological mechanisms remain uncertain. Longitudinal studies with larger samples and repeated biomarker measurements are needed to clarify the directionality, robustness, and mechanisms underlying these associations.

## Figures and Tables

**Table 1:** Comparison of sociodemographic and lifestyle characteristics, and A/ASP and DEBQ scores between groups.

Variables	Groups		*p*
	CG (*n* = 48)	OB (*n* = 51)	
Age (years)	14.99 ± 1.48	14.55 ± 1.76	0.188
Height (cm)	164.07 ± 7.27	164.21 ± 7.78	0.930
Sex, *n* (%)			
Female	34 (70.8)	33 (64.7)	0.515
Male	14 (29.1)	18 (35.2)	
Screen time, *n* (%)			
≤3 h/day	26 (54.2)	17 (33.3)	0.059
>3 h/day	22 (45.8)	34 (66.7)	
Activity level, *n* (%)			
Inactive/less active	9 (18.8)	31 (60.8)	**<0.001**
Active/very active	39 (81.3)	20 (39.2)	
Late and/or irregular sleep, *n* (%)			
Absent	42 (87.5)	45 (88.2)	0.999
Present	6 (12.5)	6 (12.5)	
Sleep duration, *n* (%)			
≤8 h	33 (68.8)	31 (60.8)	0.536
>8 h	15 (31.3)	20 (39.2)	
Maternal education, *n* (%)			
Primary or less	18 (37.5)	25 (49.0)	0.097
Secondary	20 (41.7)	11 (21.6)	
High school or above	10 (20.8)	15 (29.4)	
Paternal education, *n* (%)			
Primary or less	15 (31.3)	18 (35.3)	0.129
Secondary	19 (39.6)	11 (21.6)	
High school or above	14 (29.2)	22 (43.1)	
Income status, *n* (%)			
Expenses > income	4 (8.3)	7 (13.7)	0.679
Income = expenses	25 (52.1)	26 (51.0)	
Income > expenses	19 (39.6)	18 (35.3)	
**A/ASP (mean ± SD)**			
Low Registration	31.54 ± 4.32	32.18 ± 5.64	0.533
Sensation Seeking	43.38 ± 7.69	44.90 ± 7.72	0.327
Sensory Sensitivity	38.44 ± 7.66	40.73 ± 9.05	0.179
Sensation Avoiding	39.10 ± 5.71	38.94 ± 4.09	0.870
**DEBQ (mean ± SD)**			
Emotional Eating	27.69 ± 11.74	31.61 ± 11.96	0.103
External Eating	21.46 ± 9.47	25.69 ± 8.81	**0.024**
Restrained Eating	31.35 ± 7.43	30.25 ± 8.93	0.508

Values are presented as mean ± SD or *n* (%). Independent-samples *t*-test and Chi-square test were applied as appropriate. Significant *p*-values are shown in bold.

**Table 2:** Distribution of A/ASP Quadrant Scores in Adolescents with Obese and Control Groups.

A/ASP Quadrants	Group	Much Less Than Most People *n* (%)	Less Than Most People *n* (%)	Similar to Most People *n* (%)	More Than Most People *n* (%)	Much More Than Most People *n* (%)
Low Registration	OB	0 (0)	10 (19.6)	39 (76.5)	2 (3.9)	0 (0)
	CG	0 (0)	8 (16.7)	39 (81.3)	1 (2.1)	0 (0)
Sensation Seeking	OB	1 (2.0)	12 (23.5)	37 (72.5)	1 (2.0)	0 (0)
	CG	1 (2.1)	18 (37.5)	28 (58.3)	1 (2.1)	0 (0)
Sensory Sensitivity	OB	0 (0)	1 (2.0)	26 (51.0)	13 (25.5)	11 (21.6)
	CG	0 (0)	0 (0)	33 (68.8)	8 (16.7)	7 (14.6)
Sensation Avoiding	OB	0 (0)	0 (0)	31 (60.8)	20 (39.2)	0 (0)
	CG	0 (0)	0 (0)	34 (70.8)	14 (29.1)	0 (0)

Note. Data are presented as *n* (%).

**Table 3:** Correlations between A/ASP quadrants and DEBQ subscales in adolescents (*n* = 99).

A/ASP Quadrants	Emotional Eating (DEBQ)	External Eating (DEBQ)	Restrained Eating (DEBQ)
Low Registration	r = 0.151	r = −0.021	r = 0.075
	*p* = 0.137	*p* = 0.835	*p* = 0.463
	*p* ^#^ = 0.284	*p* ^#^ = 0.835	*p* ^#^ = 0.695
Sensation Seeking	r = 0.063	r = 0.178	r = 0.103
	*p* = 0.533	*p* = 0.077	*p* = 0.309
	*p* ^#^ = 0.711	*p* ^#^ = 0.284	*p* ^#^ = 0.530
Sensory Sensitivity	r = 0.442 **	r = 0.242 *	r = 0.167
	*p* < 0.001	*p* = 0.016	*p* = 0.098
	*p* ^#^ < 0.001	*p* ^#^ = 0.096	*p* ^#^ = 0.284
Sensation Avoiding	r = −0.025	r = 0.149	r = −0.032
	*p* = 0.804	*p* = 0.142	*p* = 0.755
	*p* ^#^ = 0.835	*p* ^#^ = 0.284	*p* ^#^ = 0.835

Note. Correlations were computed using Pearson correlation analysis, ** *p* < 0.01, * *p* < 0.05, *p* ^#^ values were adjusted using the Benjamini–Hochberg (BH) false discovery rate correction.

**Table 4:** Regression coefficients for the effects of BMI, sex, PA, leptin, ghrelin, and cortisol on emotional eating (hierarchical multiple regression).

Variables	β	SE	β *	*p*	R^2^	R^2^ *	Tolerance	VIF
Block 1								
Constant (β_0_)	13.72	7.35	-	0.066	0.10	0.07		
BMI (β_1_)	0.55	0.23	0.29	0.018			0.73	1.37
Sex (β_2_)	−5.76	2.68	−0.22	0.035			0.99	1.01
PA (β_3_)	4.40	2.98	0.18	0.143			0.73	1.37
Block 2								
Constant (β_0_)	12.28	7.70	-	0.114	0.11	0.07		
BMI (β_1_)	0.57	0.23	0.30	0.016			0.73	1.38
Sex (β_2_)	−5.60	2.70	−0.22	0.042			0.98	1.02
PA (β_3_)	4.48	2.99	0.18	0.138			0.73	1.37
Leptin	−0.01	0.01	−0.07	0.535			0.96	1.04
Ghrelin	0.01	0.01	0.07	0.511			0.98	1.02
Cortisol	2.18	6.34	0.03	0.732			0.99	1.01

Notes. β = unstandardized beta coefficient; SE = standard error of β; β * = standardized beta coefficient; *R*^2^ = coefficient of determination; R^2^ * = adjusted R^2^; VIF = variance inflation factor.

**Table 5:** Regression coefficients for the effects of BMI, sex, PA, leptin, ghrelin, and cortisol on external eating (hierarchical multiple regression).

Variables	β	SE	β *	*p*	R^2^	R^2^ *	Tolerance	VIF
Block 1								
Constant (β_0_)	7.67	5.31	-	0.153	0.10	0.07		
BMI (β_1_)	0.51	0.17	0.38	0.003			0.73	1.37
Sex (β_2_)	−0.34	1.94	−0.02	0.863			0.99	1.01
PA (β_3_)	3.28	2.15	0.18	0.131			0.73	1.37
Block 2								
Constant (β_0_)	6.15	5.54	-	0.270	0.11	0.07		
BMI (β_1_)	0.53	0.17	0.39	0.002			0.73	1.38
Sex (β_2_)	−0.16	1.95	−0.01	0.934			0.98	1.02
PA (β_3_)	3.35	2.15	0.19	0.123			0.73	1.37
Leptin	−0.01	0.01	−0.15	0.157			0.959	1.04
Ghrelin	0.01	0.01	0.10	0.337			0.98	1.02
Cortisol	0.63	5.00	0.01	0.900			0.99	1.01

Notes. β = unstandardized beta coefficient; SE = standard error of β; β * = standardized beta coefficient; *R*^2^ = coefficient of determination; R^2^ * = adjusted R^2^; VIF = variance inflation factor.

**Table 6:** Regression coefficients for the effects of BMI, sex, PA, leptin, ghrelin, and cortisol on restrained eating (hierarchical multiple regression).

Variables	β	SE	β *	*p*	R^2^	R^2^ *	Tolerance	VIF
Block 1								
Constant (β_0_)	34.47	5.11	-	<0.001	0.018	0.017		
BMI (β_1_)	−0.10	0.16	−0.08	0.534			0.73	1.37
Sex (β_2_)	−1.88	1.86	−0.11	0.317			0.99	1.01
PA (β_3_)	−0.87	2.07	−0.05	0.674			0.73	1.37
Block 2								
Constant (β_0_)	34.52	5.36	-	<0.001	0.018	0.029		
BMI (β_1_)	−0.10	0.16	−0.08	0.536			0.70	1.42
Sex (β_2_)	−1.88	1.88	−0.11	0.320			0.99	1.01
PA (β_3_)	−0.87	2.08	−0.05	0.676			0.72	1.39
Leptin	0.00	0.01	0.05	0.679			0.96	1.04
Ghrelin	0.00	0.01	0.01	0.974			0.96	1.04
Cortisol	−5.12	4.55	−0.12	0.263			0.99	1.01

Notes. β = unstandardized beta coefficient; SE = standard error of β; *β* * = standardized beta coefficient; *R*^2^ = coefficient of determination; **R^2^** * = adjusted R^2^; VIF = variance inflation factor.

## Data Availability

The data presented in this study are available on reasonable request from the corresponding author. The data are not publicly available due to privacy and ethical restrictions.

## Supplementary Materials

The following supporting information can be downloaded at: https://www.mdpi.com/article/10.3390/nu17243923/s1, Table S1: Comparison of DEBQ Eating Behavior Scores by Sex; Figure S1: Eating behaviors by sex in the obese (OG) and control (CG) groups; Table S2: Plasma Leptin, Plasma Ghrelin, and Salivary Cortisol Levels in Obese and Control Groups.

## Abbreviations

The following abbreviations are used in this manuscript:

BMI	Body Mass Index
WHO	World Health Organization
A/ASP	Adolescent/Adult Sensory Profile
DEBQ	Dutch Eating Behavior Questionnaire
