# Supplementary material for: The Relationship of Sensory Profiles and Peripheral Biomarkers with Obesity and Eating Styles in Adolescence
Source: Nutrients. 2025 Dec 15;17(24):3923. doi: 10.3390/nu17243923 (PMC12736243; doi:10.3390/nu17243923)
Supplement: Supplementary file 1 [file nutrients-17-03923-s001.zip › nutrients-4011188-supplementary.pdf]

SUPPLEMENTS

Table S1. Comparison of DEBQ Eating Behavior Scores by Sex.

DEBQ	**Female** (*n*=67)	**Male** (*n*=32)	
Emotional Eating	31.51±12.33	25.94±10.32	**0.021**
External Eating	23.46±8.85	24.0±10.4	0.802
Restrained Eating	31.04±8.45	30.25±7.78	0.646

Data are presented as mean ± standard deviation (SD). Independent samples t-test was used for group comparisons.

Figure S1. Eating behaviors by sex in the obese (OG) and control (CG) groups.

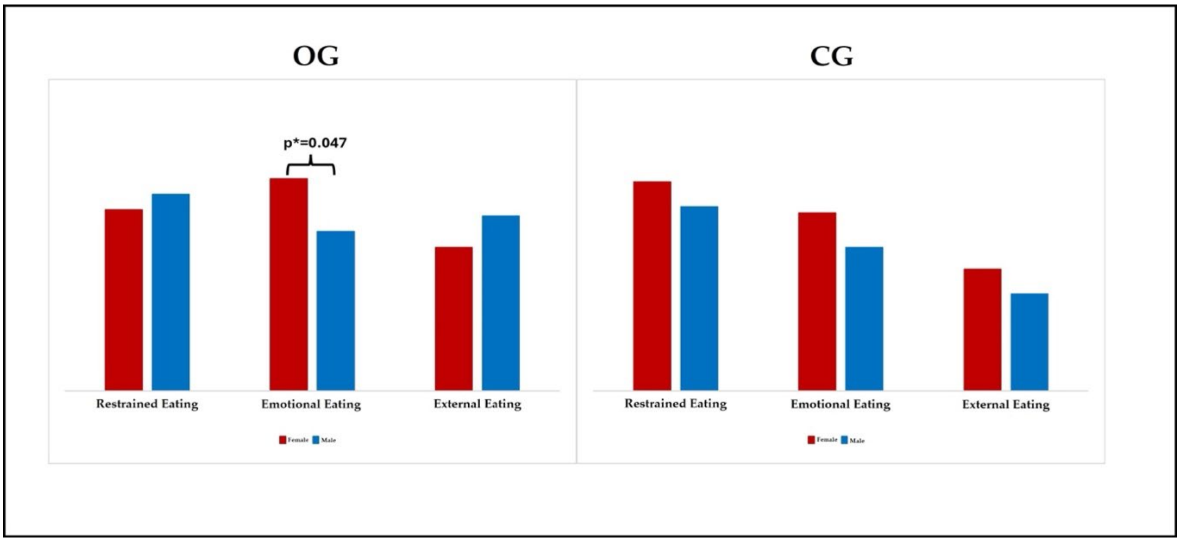

Table S2. Plasma Leptin, Plasma Ghrelin, and Salivary Cortisol Levels in Obese and Control Groups.

	**Groups**		***p***
	**CG**	**OG**	
Plasma Leptin (pg/mL)	*n*=44<br>245.5(177.0-387.8)	*n*=44<br>275.0(176.8-495.5)	0.679
Plasma Ghrelin (pg/mL)	*n*=44<br>1927.0(679.8-3002.0)	*n*=44<br>1177.0(483.3-2079.0)	0.108
Salivary Cortisol (µg/dL)	*n*=48<br>0.2(0.2-0.3)	*n*=51<br>0.2(0.1-0.3)	0.774

Data are presented as median (IQR, Q1–Q3). Mann–Whitney U test was used for comparisons.
